# Kinetics of circulating cell-free DNA for biomedical applications: critical appraisal of the literature

**DOI:** 10.4155/fsoa-2017-0140

**Published:** 2018-02-23

**Authors:** Sonia Khier, Laura Lohan

**Affiliations:** 1 Department of Pharmacokinetics, Univ Montpellier, Montpellier, France; 2 IRCM, Inserm, Univ Montpellier, ICM, Montpellier, France

**Keywords:** biomarkers, cell-free DNA, half-life, personalized medicine, pharmacokinetics

## Abstract

Circulating cell-free DNA is considered as one of the major breakthroughs in the field of innovative diagnosis, used as a liquid biopsy. The kinetic parameters of a biomarker are mandatory to assess its usefulness as a diagnostic tool. Obtaining precise mathematical values for the kinetic parameters (e.g., half-life) is then crucial because it could be used for therapeutic monitoring as a prognostic factor. However, little is known about the intrinsic properties of circulating cell-free DNA, more especially, its kinetic properties within the organism. We summarized the basic principles that may affect the kinetics of circulating cell-free DNA within the organism in the light of biological and clinical evidence. We also meta-analyzed the reported data in the literature and the methodologies that have been used to study the kinetic parameters of human circulating cell-free DNA *in vivo*.

## Lay abstract

Circulating cell-free DNA as a biomarker was a major breakthrough in the field of diagnostics. Understanding the kinetic parameters of a biomarker is mandatory to assess its usefulness as a diagnostic tool, especially for therapeutic monitoring. However, at the present time little is known about its kinetic properties within the organism. This review provides an overview of the basic principles that may impact the kinetics of cell-free DNA within the organism and analyzes the reported data thus far.

Circulating cell-free DNA (cfDNA) is derived from cells in the blood following different mechanisms such as cell death, active secretion, ETosis, phagocytosis, autophagocytosis, etc. [1,2]. Increases in cfDNA levels is described in various pathologic conditions such as cancer, sepsis, autoimmune diseases and in particular, physiological states such as pregnancy or intense physical exercise. As a result, the cfDNA level is studied as a potential biomarker for early diagnosis, diagnosis and prognosis. As a potential biomarker, it is crucial to characterize the intrinsic kinetics properties of cfDNA within the organism, especially when using cfDNA analysis as longitudinal diagnostic tool for monitoring the course of treatment. As an endogenous biological product, cfDNA may follow the physiological distribution (in tissues and physiological fluids) and elimination processes. Kinetic parameters are established when analyzing the evolution of blood, serum or plasma concentrations of a substance over time. Thus, the evolution of the cfDNA concentration over time reflects those processes. Intra- or inter-individual variability due to particular physiological or physiopathological conditions may affect the level of cfDNA. Obtaining precise mathematical values for the kinetic parameters is then crucial because these values could be the reflection of the progression or the regression of a pathology, yet also be a prognostic factor for treatment. While several works were interested in the biodistribution and degradation of genomic DNA throughout the organism, few works have been interested in determining the biodistribution and elimination of cfDNA *stricto sensu*.

## Physiological & pathological processes responsible for the cfDNA rate

### cfDNA release into the blood

**Figure 1.**
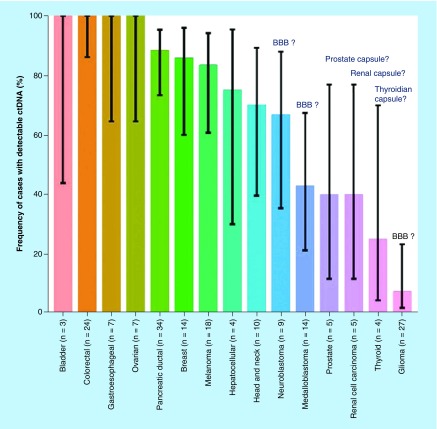
**Physiological barriers seem to affect the distribution of tumor-derived cell-free DNA.** BBB: Blood–brain barrier; ctDNA: Circulating tumor DNA. Reproduced with permission from [4] © The American Association for the Advancement of Science (2018).

cfDNA is released into the blood following cell death mechanisms and/or active secretion. During pregnancy, in addition to their own cfDNA, fetal cfDNA (f-cfDNA) circulates in the blood of pregnant women [3]. In cancer patients, there is a fraction of cfDNA derived from the tumor. It appears that the level of cfDNA in cancer patients depends on the location of tumor. Currently, colon, gastroduodenal tract, breast, pancreas, liver and skin cancers are clearly identified as releasing large amounts of cfDNA within blood while glioma and thyroid tumors release small amounts of cfDNA [4]. We can hypothesize that cfDNA does not cross the blood–brain barrier or the capsule protecting organs such as thyroid, prostate or kidneys (Figure 1) but can easily cross placental barrier. Much like drugs, these physiological barriers may affect the distribution (and the rate) of cfDNA throughout the organism.

### cfDNA distribution throughout the organism

It is well known that cfDNA is present in human plasma as well as in other biological fluids (urine, feces, cerebrospinal fluid and milk [2]). cfDNA is also present in interstitial spaces [5]. This can be explained by the fact that all cells release cfDNA. However, cfDNA has been shown to be an intercellular messenger and this suggests that it can travel from one site to another within the organism [1].

#### Evidence based on cfDNA messenger capacity

M Stroun, P Anker and P Gahan were the first to show the DNA mobility within an organism or between organisms [6–11]. They hypothesized that nucleic acids were active carriers of the genetic information between cells [12]. Various uptake mechanisms can be hypothesized: they can be mediated by extracellular receptors for cfDNA such as TLR9 or HGMB1 [13], with the complexes formed between cfDNA and plasma proteins favoring their internalization using protein receptors [14]. In addition, nucleosomes have been shown to be able to cross the cellular membrane [15], while exosomes can undergo endocytosis [16]. Nevertheless, all those observations are based on *in vitro* experiments or *in vivo* experiments using cfDNA derived from *in vitro* experiments.

#### Evidence based on identified cfDNA structures

A significant amount of cfDNA has been shown to be associated with the extracellular surface of blood cells (cell-surface-bound cfDNA [csb-cfDNA]), both erythrocytes and leukocytes due to the presence of nucleic acid-binding proteins [17,18]. The cfDNA distribution space could also include the lymphatic system, implying a volume of distribution greater than blood. In addition, cfDNA structures seem heterogeneous (ssDNA or dsDNA fragments from dozen to thousands bp, macromolecular structures such as mono- or oligo-nucleosomes, neutrophil extracellular traps [NETs], nucleolipidoproteic complexes or as microvesicular structures such as microparticles [200–1000 nm] or apoptotic bodies [1–5 μM] [19,20]). All these cfDNA structures can be present on a cell’s surface at the same time. The binding can be reversible, saturable and can also promote internalization of DNA in blood cells [20]. We can hypothesize that free cfDNA or microvesicles could pass through the sinusoidal capillaries to reach other organs such as bone, liver and spleen.

**Figure 2.**
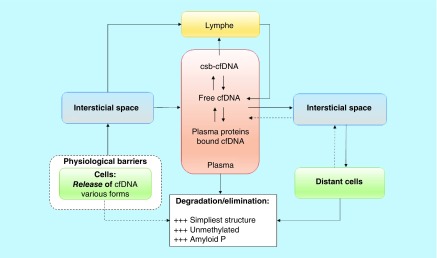
**Illustration of cfDNA distribution within the organism based upon identified processes and raised questions.** cfDNA: Cell-free DNA; csb-cfDNA: Cell-surface-bound cfDNA.

Altogether, these evidences reveal that cfDNA is distributed within the organism subsequent to its release within the blood (Figure 2).

### cfDNA elimination process

#### cfDNA elimination pathway

Data obtained from animal studies are difficult to extrapolate to humans as the DNA used in these studies is injected, highly purified and comes from different species. However, all these preclinical experiments provide a preliminary insight into the elimination process and show that DNA is rapidly degraded by nucleases present in the blood and rapidly eliminated by the liver and kidneys [21–23]. Lo *et al.* [24] subsequently performed an *in vitro* assay to experiment nucleases’ role on f-cfDNA clearance from maternal plasma. They concluded that plasma nuclease only plays a partial role in the removal of f-cfDNA in most subjects, and that other organ systems are involved. The experiments carried out by Yu *et al.* [25] led to similar conclusion derived from *in vivo* experiments on pregnant women: neither plasma nucleases nor the kidney were the major routes for f-cfDNA elimination. While we cannot confirm the role of DNase activity and renal excretion in the human cfDNA elimination process, we can nevertheless expect them to play a role in the elimination pathway, even if it is minor. As cfDNA can be excreted in urine, recent works have explored detection of cfDNA in urine as a potential ‘liquid biopsy’ [26–28].

#### Factors affecting cfDNA elimination

As already mentioned above, while the eclectic structure of cfDNA present in human blood has an impact on its distribution, it can also have an impact on its elimination. In a pathological context, such as systemic lupus, the presence of a large amount of complex (cfDNA with monoclonal autoantibody) in plasma could have an impact on the half-life (HL) elimination value by decreasing the clearance resulting from a problem of DNases accessibility. Complex cfDNA structures such as nucleosomes, mono- or oligo-nucleosomes, virtosomes, by preventing access to DNases, could in the same way complicate enzymatic activity by nucleases. A lack or diminution of DNase activity could also influence the cfDNA elimination rate. This has been pointed out within a pathological context with systemic lupus [29,30] and cancer. The DNA hydrolyzing activity reveals a low activity in cancer patients and a high blood plasma activity in healthy donors [31].

At last, the third factor influencing elimination rate is the binding to plasma proteins. The human serum albumin binding was shown to result in long-term cfDNA plasma circulation [32]. The result is an increase in the elimination HL. Skvortsova *et al.* [33] postulated that an efficient binding of methylated DNA with blood proteins could explain a slower degradation of methylated DNA compared with an unmethylated DNA. On the other hand, studies revealed that serum amyloid P-component might be involved in one of the mechanisms responsible for effective cfDNA clearance inasmuch as it binds cfDNA [34]. It seems that the involvement of plasma proteins in the elimination process depends on the class of plasma proteins interacting with cfDNA.

Different factors (DNA-hydrolyzing enzymes, the structure of cfDNA and protein interactions in blood) can affect the elimination process. Impact of each factor on elimination speed cannot be quantified yet. Hence, determining a global kinetic parameter remains the best way to study the various rates of cfDNA elimination.

## Kinetic parameters

**Figure 3.**
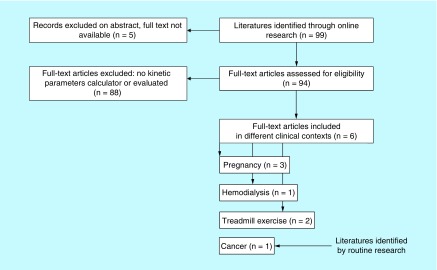
**Flow chart of the included studies in the meta-analysis.**

Of the 99 complete texts potentially eligible for meta-analysis using keyword research, only six original articles [24–25,35–38] were selected (Figure 3) and analyzed, assessing a kinetic parameter related to *stricto sensu* human cfDNA (e.g., endogenous human product). We also included the original article found through a routine review of cancer literature that led us to make this review [39]. The main kinetic parameter investigated is the HL. In Table 1 we summarize results and design trials.

**Table 1.** **Half-life of circulating cell-free DNA calculated in different contexts.**

**N patients^†^**	**Half-life value – dispersion parameter**	**N sample points for calculation^‡^ [first point; last point]**	**Calculation method^§^**	**Total of sample points obtained per patient^¶^ between [t_0_; t_f]_**	**Study**	**Ref.**
**Treadmill exercise (q-PCR : *MSTN*)**						

3	16 min (mean) – range: [9–23]	NA [NA; NA]	NA	5 [End of exercise: t_0_ + 30 min]	Beiter *et al.*	[37]

**Hemodialysis context (purified DNA radiolabeled)**						

15	4 min (mean) – SD = ± 1.2 min	2 [Mean absolute concentration at t_0;_ C_(t = 5 min)_ or C_(t = 10 min)]_	Least squares Data expressed as a fraction of the zero concentration	2 or 3 [End of hemodialysis; t_0_ + 10 min]	Rumore *et al.*	[35]

**Pregnancy – fetal ccfDNA (q-PCR : *SRY*)**						

8	16.3 min (mean) – range: [4–30]	n = NA [Peak concentration; NA]	Mean time taken to reduce the peak plasma fetal DNA concentration by 50%	7 [Cesarean; t_0_ + 120 min]	Lo *et al.* 1999	[24]

7 pre-eclampsie	114 min (median) – range: [46–210] Interquartil: [81–168]	n = 3–7 [Peak concentration^#^; first non-zero trough^##^]	Log-linear regression (SigmaStat 2.0)	8 [Cesarean; t_0_ + 360 min]	Lau *et al.* 2002	[36]

9 control	28 min (median) – range: [7–114] Interquartil: [16–47]					[36]

8	0.9 h (mean) – range: [0.6–1.2]	n = 4–6 (ns) [Peak concentration (ns); 120 min]	Log-linear regression	6–7 [Cesarean; t_0_ + 24 h] cases 1–3 [Cesarean; t_0_ + 120 min] cases 4–5 [Cesarean; t_0_ + 18 h] cases 6–8	Yu *et al.* 2013	[25]

3 *(among the 8)*	12.6 h (mean) – range: [4.2–18.1]	NS = 2–3 (ns) [6 h; 18 h] cases 6–8				[25]

**Colorectal tumor (q-PCR: *TP53*).**						

1	114 min – NA	n = 4 [C_Day1_; C_Day3_]	Non linear regression (LM algorithm)	8 [Surgical resection; t_0_ + ∼350 days]	Diehl *et al.* 2008	[39]

For all the studies, plasma was the sample matrix.

^†^Number of patients included in the analysis.

^‡^Number of plasma samples used for calculation of half-life.

^§^Method used to evaluate half-life.

^¶^Total number of samples obtained from patient along the study, baseline included.

^#^5–45 min post-delivery.

^##^ 45–360 min post-delivery.

cfDNA: Circulating cell-free DNA; NA: Not available; ns: Not specified in the text, the values were evaluated from concentration–time curves; q-PCR: Quantitative PCR; SD: Standard deviation; t_0_: Beginning of the kinetics, from this time a decline of cfDNA concentration is expected; t_f_: Time of the last sample obtained during assay.

### Treadmill exercise

Intense exercise has been reported to increase cfDNA plasma concentrations [40,41]. As inflammatory markers are enhanced following resistance exercise, Fatouros *et al.* [42] suggest that cfDNA in plasma might be a sensitive marker for exercise-induced inflammation. Thus, physical exercise was used as a model to study the evolution of cfDNA within the organism. Beiter *et al.* [37] and Breitbach *et al.* [38] studied a deliberately induced increase of cfDNA in controlled conditions. Samples were collected from healthy subjects (athletes) during a treadmill exercise. Beiter *et al.* analyzed nuclear cfDNA (88 bp fragment of the chromosomal myostatin gene locus, *MSTN*) and mitochondrial cfDNA (85 bp fragment of the mitochondrial genome) from venous blood sample by quantitative (q)-PCR. The blood samples were obtained from three individuals during and after the treadmill exercise. A mean HL of 16 min: [range 9–23 min] was obtained. Breitbach *et al.* applied a new analytic procedure requiring a minimal volume of unpurified plasma for quantification. Capillary blood was collected from the fingertip from 26 athletes (amplification of a 90- and a 222-bp multilocus *L1PA2* sequence by q-PCR). During discussion, the authors assumed that a mean HL rate of approximately 15 min could be verified.

### Hemodialysis

DNA is considered to be the antigenic precursor of DNA–anti-DNA immune complexes. The DNA elimination rate would be expected to affect the formation and pathogenicity of these immune complexes. Rumore *et al.* [35] chose hemodialysis as a model of cfDNA release and proposed determining the elimination rate. During hemodialysis, cfDNA may be released from cells trapped within the dialysis coil. Since that release presumably ceases with the termination of the procedure, it offers an opportunity to estimate the elimination rate of cfDNA in humans with controlled conditions and minimize the interindividual variability of the release (no traumatic release). Blood samples were obtained during the 10-min posthemodialysis from 15 patients undergoing routine hemodialysis. A mean HL of 4 min was obtained.

### Cancer

Blood concentration of cfDNA proved to be high under several pathological conditions such as cancer. Diehl *et al.* [39] applied a highly sensitive approach to quantify tumor cfDNA in plasma samples from subjects undergoing multimodality therapy for colorectal cancer. They proposed to use a cfDNA concentration to monitor the tumor dynamics of patients with metastatic colorectal cancer under surgery and chemotherapy. A total of 162 plasma samples from the 18 subjects were collected before and after surgery and a q-PCR on *APC*, *KRAS*, *PIK3CA* or *RAS* was realized. A HL of 114 min was obtained from one subject among 18.

### Pregnancy

The population within which the kinetic parameter of human cfDNA has been the most studied is that of pregnant women. Since the discovery of f-cfDNA in the maternal blood [3], a number of clinical applications involving the use of this f-cfDNA have been proposed [43]. These include prenatal diagnostic screening and could also serve as a prognostic biomarker for maternal pathologies. Lo *et al.* [24] were the first to determine a quantitative kinetic parameter of f-cfDNA from maternal plasma. After delivery (cesarean) maternal peripheral blood was collected at different times from eight women (free of any medical disease or any antenatal complications). f-cfDNA was quantified by q-PCR, targeting a sequence of 198 bp on *SRY* (only male fetuses were selected). The mean time taken to reduce the peak plasma f-cfDNA concentration by 50% was 16.3 min (range: 4–30 min) representing mean plasma HL. While the f-cfDNA elimination process from the maternal plasma may appear to be rapid, a number of pathological conditions associated with damage to and dysfunction of the kidneys have been associated with the impaired clearance of f-cfDNA. For this reason, Lau *et al.* [36] proposed describing the kinetics of f-cfDNA in the plasma of seven pre-eclamptic women versus nine control women in the same condition as Lo *et al.* (post-cesarean sampling, q-PCR, *SRY*). As the f-cfDNA concentration in maternal plasma is higher in pre-eclamptic women than in pregnant control women of the same gestational age, they hypothesized that a slower maternal elimination process could explain the different level of f-cfDNA concentration. A median HL of 28 min (range: 7–114 min) was evaluated for the control group and a median HL of 114 min [range 46–210 min] for the pre-eclamptic group. Yu *et al.* [25] proposed identifying the major f-cfDNA elimination route (renal or nucleases elimination) using a precisely quantified f-cfDNA (*SRY*). For eight women with uncomplicated singleton pregnancies, the number of samples was sufficient to determine a concentration versus time curve. A median HL of 0.9 h (range: 0.6–1.2 h) was evaluated. But for three women among the eight, the sampling protocol was different and late sampling points have been integrated in the concentration versus time curve. For these three women, two phases of decreased concentration were observed, leading to a median HL evaluation for the ‘rapid phase’ of 0.9 h (range: 0.6–1.2 h) and an HL for the ‘slow phase’ of 12.6 h (range: 4.2–18.1 h).

## Discussion

### Assessment of kinetic parameters

A parallel can be drawn between pharmacokinetic (PK) parameters obtained for a drug and the kinetic parameters of a biomarker/endogenous product. This is because these entities can pursue the same physiological biodistribution and elimination stages and because the parameters used to describe the kinetics are the same. The methodologies used to obtain PK parameters are presented as supplementary data.

### Physiological cfDNA release

Beiter *et al.* [37] studied the time–concentration course of cfDNA in controlled conditions (healthy athletes) during a treadmill exercise. These two cases are particularly interesting because they permit to study cfDNA under basal conditions (no pathologies) and its release with a minimal source of variability. Despite these conditions, the concentrations versus time profile is very different from one subject to another (see multipanel curves in Supplementary Data). It is worthwhile noting that plasma mitochondrial cfDNA concentrations were not affected by the treadmill exercise. For the kinetic protocol, series of blood samples from three participants were collected without any further information concerning the method or study design used for the kinetic analysis. Within the same context (treadmill exercise), Breitbach *et al.* [38] discuss a rapid increment of cfDNA concentrations and that “*in 50% of the subjects in the present study, the mean HL rate of approximately 15 min could be verified*”, for a total of 26 subjects included in the study, without any further information concerning the calculation method or study design for the kinetic analysis. It would be interesting to obtain more accurate results, and more information about design protocol and methodology.

Rumore *et al.* [35] chose the cfDNA released into the plasma during the hemodialysis course as a model. A question was raised concerning the methodology. Blood samples were collected from 15 patients, with 11 blood samples taken at 5 min, 13 blood samples at 10 min and we assumed 15 blood samples at t_0_. The slope was calculated with the mean absolute concentration at t_0_ (from the 15 patients) and t_5 min_ or t_10 min_ (see multipanel curves in Supplementary Data). The slope could be evaluated with just two values, but this does not match the practices generally used in PK analyses which require a minimum of three values to calculate a slope. Other biases are explained by the author, such as the selection of patients included in the study being dependent on the concentration at t_0_. The choice of hemodialysis is interesting because it permits elimination to be studied without provoking a peak concentration caused by a traumatic process (pathology, surgery or exercise). However, knowing that the kidney is involved in the elimination of cfDNA (even as a minor route), this implies that the physiopathological status of patients (renal failure) has an impact on the elimination rate and the evaluated HL. In the PK field, patients with disorders concerning elimination pathways are excluded from early studies to study kinetics in a standardized situation.

### Interindividual variability

Pathological states can influence the cfDNA concentration. Csb-cfDNA concentrations and content are both altered in cancer patients. The cell-free to csb-cfDNA ratio in blood depends on the condition of the donor. While the majority of cfDNA from healthy donors is linked to cells, cancer patients possess an altered distribution of cfDNA that depends on type and location of the tumor [20]. As previously mentioned, plasma DNase reveals a lower hydrolyzing activity in cancer patients than in healthy donors [31]. In addition, the protein binding of cfDNA to plasma proteins greatly influences its distribution as protein concentration can be affected by the physiopathology state of donors (hypo-hyper albuminaemia, systemic lupus erythematosus, etc.). These results show different sources of variability of concentration cfDNA.

The results of Diehl *et al.* [39] highlight the problem of variability in a particular context. They worked on the detection of a cfDNA-specific gene mutation that could be used as a useful biomarker in cancer detection, monitoring and prognostics. The aim of the study was not to determine kinetic parameters and, consequently, the design was not optimized for that purpose. The HL determination was set for one patient, having a smooth concentration curve shape. Because the interindividual variability was too high, calculating an HL for the 18 patients included in the study proved difficult (see multipanel curves in Supplementary Data). The causes of variabilities in the curves shape mainly result from the various therapy sources (chemotherapy, surgery, etc.), level of tumor resection and the condition of the patients.

It is now well known that for a same drug administered at the same dosage, the concentration curve could be different from one patient to another and for a same patient having physiopathological variations. The population pharmacokinetics approach (POPPK) is commonly used to analyze the PK parameters of drugs and also to identify and quantify the origins of interindividual variabilities [44–46]. The POPPK approach has been used for the kinetic analysis of several serum tumor biomarkers [47] and could be an interesting approach to treat the variability issue for an endogenous component [48].

### Different kinetic stages for cfDNA

Within the context of pregnancy, Yu *et al.* [25] monitored cfDNA plasma concentrations from eight subjects over a period of 18 h and more. A slope was determined by log-linear regression (we assume from the postdelivery concentration peak to the last sample concentration). The slope is expressed in HL terms by the 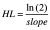 relationship, working with the hypothesis of monophasic (monoexponentiel) decrease. But for three patients with a rich data sampling from 2 to 18 h postdelivery, the shape of the concentration–time curve was modified. The log-representation of the concentration versus time was biphasic for these three patients whereas it was monophasic for the others (see multipanel curves in Supplementary Data). The biphasic curve describes two concentration rate decreases meaning different kinetic stages could be described in an independent manner.

To explain the shape of the curve, the authors hypothesized that different mechanisms could be involved in the elimination process: nucleases for rapid phase and maternal immunity and/or reticuloendothelial systems for the slow phase. However, no substantial change was apparent in the short to long f-cfDNA fragments ratio after delivery. The authors have considered two slopes (the first rapid and the second slow), both representing the elimination phase and expressing them directly in HL using the 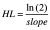 relationship. They obtained two kinetic parameters to describe the same elimination process (clearance).

**Figure 4.**
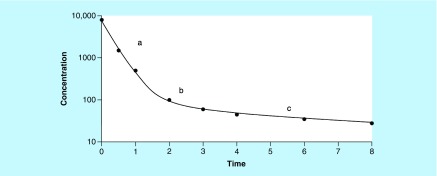
**Concentration versus time curve with a shape characteristic of two rate of concentration decrease (two-compartment model).** **(a)** Decreased concentration from the blood due to distribution and elimination process; **(b)** Inflexion point or pseudo-equilibrium state; **(c)** Decreased concentration from the blood due to elimination only (log-linear terminal phase); slope permits to calculate half-life of elimination (t_λz_).

In classical PK analysis, this biphasic shape could be assimilated to a two-compartment model (Figure 4). After the delivery-associated traumatic procedure, the first part of the curve (high slope) could describe the kinetics of the disappearance of the substance from the blood, with the disappearance due to both the distribution and elimination processes. The terminal phase (lower slope) describes the kinetics of disappearance resulting from the elimination process alone and allows the calculation of the elimination HL (t_λz_), also known as terminal HL or biological HL. The inflexion point reflects the pseudo-equilibrium stage, meaning a homogenate distribution is obtained throughout the organism. Following this reasoning, the biological HL (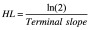) obtained by Yu *et al.* [25] is close to 12.6 h. The rate of 0.9 h corresponds to a decreasing rate prior to attaining the pseudo-equilibrium state [49] (see Supplementary Data ‘Assess pharmacokinetic parameters – Figure 2’).

### Optimal protocol design

#### Sampling design

**Figure 5.**
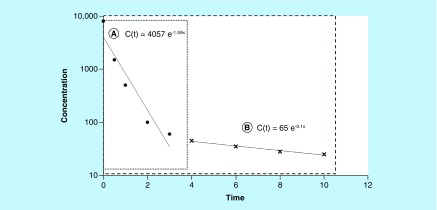
**Importance of the window blood sampling.** **(A)** Window blood sampling with t_f_ = 3 h and **(B)** window blood sampling with t_f_ = 10 h for the same entity. In the first case **(A)**, the window is too short and the elimination phase is not described alone. The slope (1.59 h-1) could be considered as the rate of elimination whereas it is the consequence of distribution and elimination process. If an optimal design is obtained (including later points ‘×’, case **[B]**), the real elimination rate is close to 0.1 h-1. t_f_: Time of the last sample obtained during assay.

As illustrated by the results of Yu *et al.*, the possibility of describing various kinetic stages depends on the blood sampling protocol. To obtain accurate kinetic parameters, concentrations must be monitored until the total elimination of the substance. The sampling interval (t_0_; t_f_; Table 1) must permit the observation of the entire kinetic process and must not be ended too rapidly (Figure 5).

In clinical or research practice, we cannot take samples until substance has been entirely eliminated (until t_∞_). When deemed appropriate, blood collection stops at a determined time (t_f_). In the best configuration, t_f_ corresponds to the time period when the concentration remains negligible, in other words, until the low limit of quantification (LLOQ). To obtain an exhaustive observation of the kinetics, sampling interval must be optimized during kinetic trials. In either case, it is necessary to obtain at least three concentrations to calculate the terminal slope. The importance and problems inherent in protocol blood sampling were underlined by Gibaldi *et al.* [50].

Yu *et al.* compared the first (rapid) phase with the HL obtained by Lo *et al.* [24] without hypothesizing or discussing the possibility that the second phase could be more representative of the elimination process. It would seem worthwhile to take at least the two HLs into account, especially if the HL could represent a biomarker for pregnancy-associated complications; in which case the second (slow phase) might be more informative. But they pointed out the fact that the discrepancies between the two results can be explained by the increased sensitivity of their detection method (leading to a bigger window sample). It is worthwhile considering the possible consequences of a longer monitoring period for pregnant women included in other studies [24,36].

Lau *et al.* [36] quantified a median HL of 114 min on seven patients (range: 46–210 min) and a median of 28 min on nine control women (range: 7–114 min) with log-linear regression. Theoretically, eight sample points should be available per patient for calculating the slope whereas for some of them, only three samples were available (see supplementary data of [36]). This is because the samples considered to compute HL are variable among the patients. The first sample point used for the log-linear regression could be 5–45 min postdelivery and the ‘cut off’ (t_f_), defined as the ‘first nonzero trough concentration’, could be 45, 60, 120 or 360 min depending on the patients.

These two limitations (limited number of sample points and restricted sampling interval) do not permit the possibility of describing a two-phase shape concentration–time curve (if any). Fatouros *et al.* [41] raised the problem of sample design in a physical exercise context. They concluded that sampling time after aseptic inflammation can lead to different conclusions regarding cfDNA responses.

#### Quality of data

Quality of data is essential to estimate accurate parameters. The more the concentrations represent ‘true concentrations’, the more the parameters will be right. To this end, the bioanalytical method must be validated for the appropriate matrix (human and/or animal plasma, blood, serum, etc.). The LLOQ should be as low as possible and the method must be sensitive and precise enough to allow the quantification of low concentrations. The latter are characteristic of the slow phase (elimination). For PK drug analysis, concentrations below the LLOQ are censored (considered as unreliable); in other words, the sample is not used for calculating parameters. In this review, we have only studied the evolution of measured cfDNA concentrations and not the bioanalytical methods used and their validation. However, we noted that Yu *et al.* [25] and Beiter *et al.* [37] were the only ones to specify a lower detection or quantification limit and did not use data providing important information [51]. Apart from under-LLOQ samples, all sample points should be used to calculate parameters and any sample point removed from the analysis needs to be justified. If we understand that there is a need to exclude some results (outliers), then this should be justified. Outliers may be considered as particular cases (rapid clearance, double peak, etc.) until further information is provided.

### What is the definition of a ‘half-life’?

‘Half-life’ is a familiar term very often used to describe the elimination of an entity, drugs or endogenous substances. A way that is often used to define or interpret biological HL is to consider HL as the time required to halve the plasma concentration. But this definition may be misleading and inappropriate because it assumes that the distribution pseudo-equilibrium is instantaneous [52]. In the PK field, the biological HL is the time required to halve the plasma concentration after reaching pseudo-equilibrium, meaning after homogenous distribution of the entity in the organism. This corresponds to the terminal log-linear curve. It is only in this context that HL can be a parameter representative of the elimination process (with one or more mechanisms included in the process). If we consider that a traumatic procedure (delivery, surgery, exercise, etc.) contributes to a sharp increase of cfDNA, we can expect plasma concentration have to reach a pseudo-equilibrium of distribution (biphasic curve). Thus, an incomplete monitoring of plasma concentration versus time or a less sensitive assay method leads to an underestimation of the terminal HL. On the contrary, different circumstances (e.g., lack of specificity of the bioanalysis method) could lead to an overestimation of HL.

## Conclusion & future perspective

Plasmatic cfDNA opens new perspectives in medical applications (prenatal diagnosis, SLE, cancer, etc.), knowing that its fluctuation rate provides interesting information for a biomarker. The results obtained to characterize the rate of elimination using calculated parameters are rare. The few reported data are discordant and no clear conclusion can be drawn. The heterogeneity of the cohorts used to determine cfDNA elimination characteristics can potentially explain the lack of concordance between the published data.

However, questions remain concerning the way to analyze the results in each context, and the definition given to the calculated parameters. Our review seems to highlight a lack of commune methodology to evaluate HL of elimination. As highlighted by a previous review concerning common biomarkers, the inconsistency of HL predictive values in various studies could be explained by the inconsistency of the methodologies used to calculate these HLs, leading to an inefficient use of reported kinetic parameters [47]. The same problem also occurs with cfDNA as a biomarker. Based on PK methodology, a kinetic analysis needs enough sample concentrations and an optimal design (accurate time sampling, suitable sampling interval) to match the parameters that are expected to be calculated. Moreover, even if HL is a standard parameter used in a biological context, we have seen that different slopes could be obtained the from C = f(t) curve. If all slopes are correct, the interpretation of the calculated parameter could be totally different. The exclusive use of HL as a reflection of cfDNA evolution over time could set aside the influence of other physiological processes that could be involved in the fluctuation rate. Other authors have worked on all the processes simultaneously (distribution and elimination) by directly exploring the area under the concentration–time curve [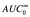] for cfDNA [53,54]. This could represent another interesting way to explore kinetics, on condition that the kinetic study is rigorously designed. In any case, absence of consensus concerning adequate methodologies used to assess kinetic biomarker parameters results in a collection of parameters with weak values, and in the future, a limited use in clinical practice.

**Executive summary**

**Physiological & pathological processes responsible for the cfDNA rate**

- Cell-free DNA (cfDNA) plasmatic rate depends on two phenomena: distribution within the organism and elimination process.
- cfDNA has been shown to be an intercellular messenger and this suggests that it can travel from one site to another within the organism.
- The elimination pathway has not been clearly identified. But plasma nucleases and renal excretion seem to play a role.
- Different factors could influence the elimination of cfDNA: structure of cfDNA, DNA hydrolyzing activity and the binding to plasma proteins.

**Kinetic parameters**

- Results concerning kinetics of ‘cfDNA’ are rare and the main parameter explored in literature is half-life.
- Half-life was obtained in different physiological or pathological circumstances: treadmill exercise, hemodialysis, pregnancy and cancer.

**Interpret the parameters**

- Physiopathological state can influence the value of half-life but the methodology used to obtain half-life value could also influence this result.
- The definition considered for half-life, protocol design and quality of data have big consequences on the half-life values obtained for circulating cfDNA used as biomarker.

**Conclusion & future perspective**

- Even if circulating cfDNA is considered to be a promising biomarker, it has not been translated into current clinical practice. This could be the result of insufficient information published concerning cfDNA kinetics.
- The exclusive use of half-life as a reflection of cfDNA evolution over time could set aside the influence of other physiological processes that could impact the fluctuation rate.
- Other possibilities to explore kinetics exist, for example, determination of the area under the concentration–time curve.
