# Supplementary material for: Kinetics of circulating cell-free DNA for biomedical applications: critical appraisal of the literature
Source: Future Sci OA. 2018 Feb 23;4(4):FSO295. doi: 10.4155/fsoa-2017-0140 (PMC5905581; doi:10.4155/fsoa-2017-0140)
Supplement: Supplementary file 1 [file fsoa-04-295-s1.pptx]

## Slide 1
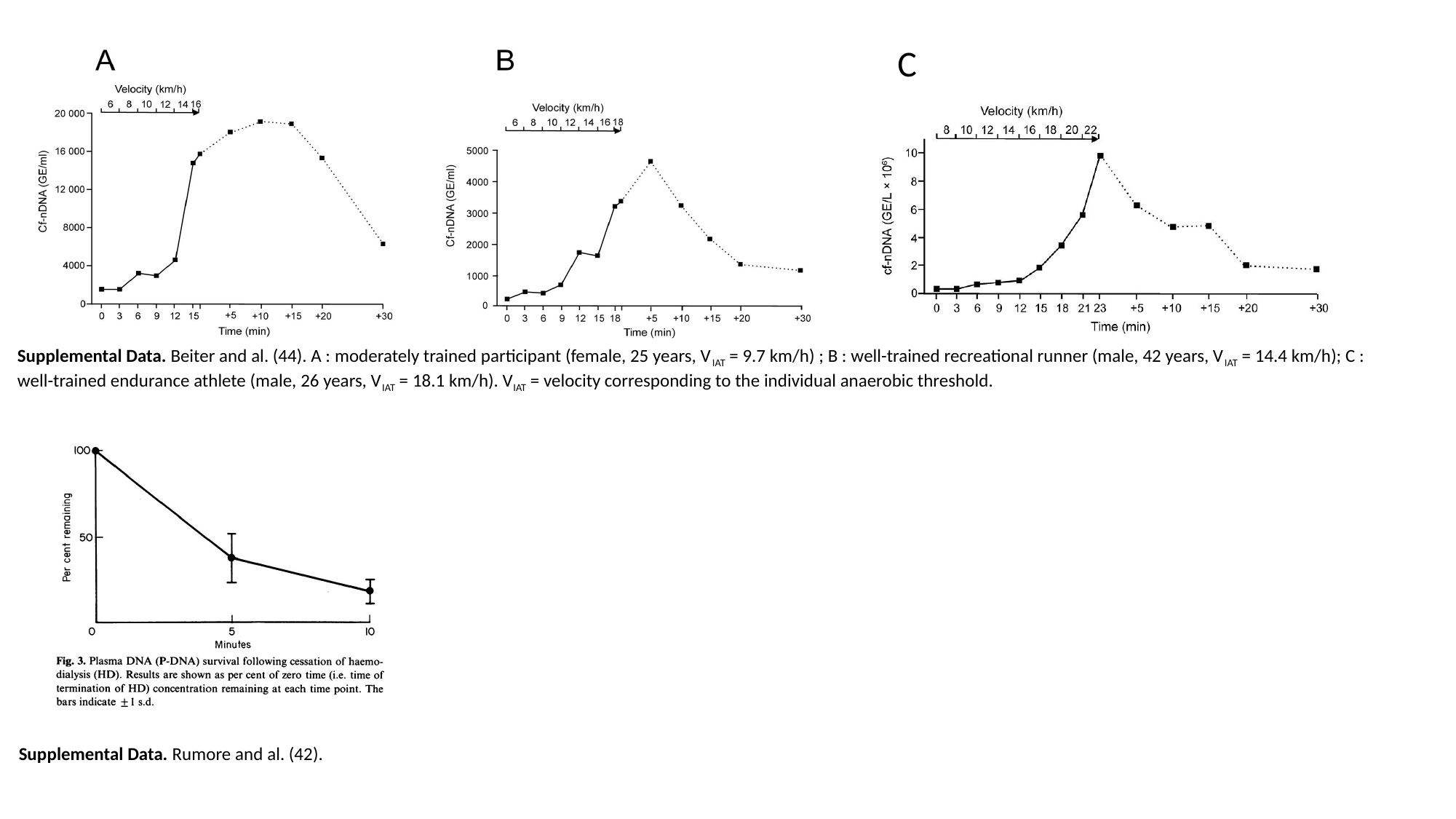

C
Supplemental Data. Beiter and al. (44). A : moderately trained participant (female, 25 years, VIAT = 9.7 km/h) ; B : well-trained recreational runner (male, 42 years, VIAT = 14.4 km/h); C : well-trained endurance athlete (male, 26 years, VIAT = 18.1 km/h). VIAT = velocity corresponding to the individual anaerobic threshold.
Supplemental Data. Rumore and al. (42).

## Slide 2
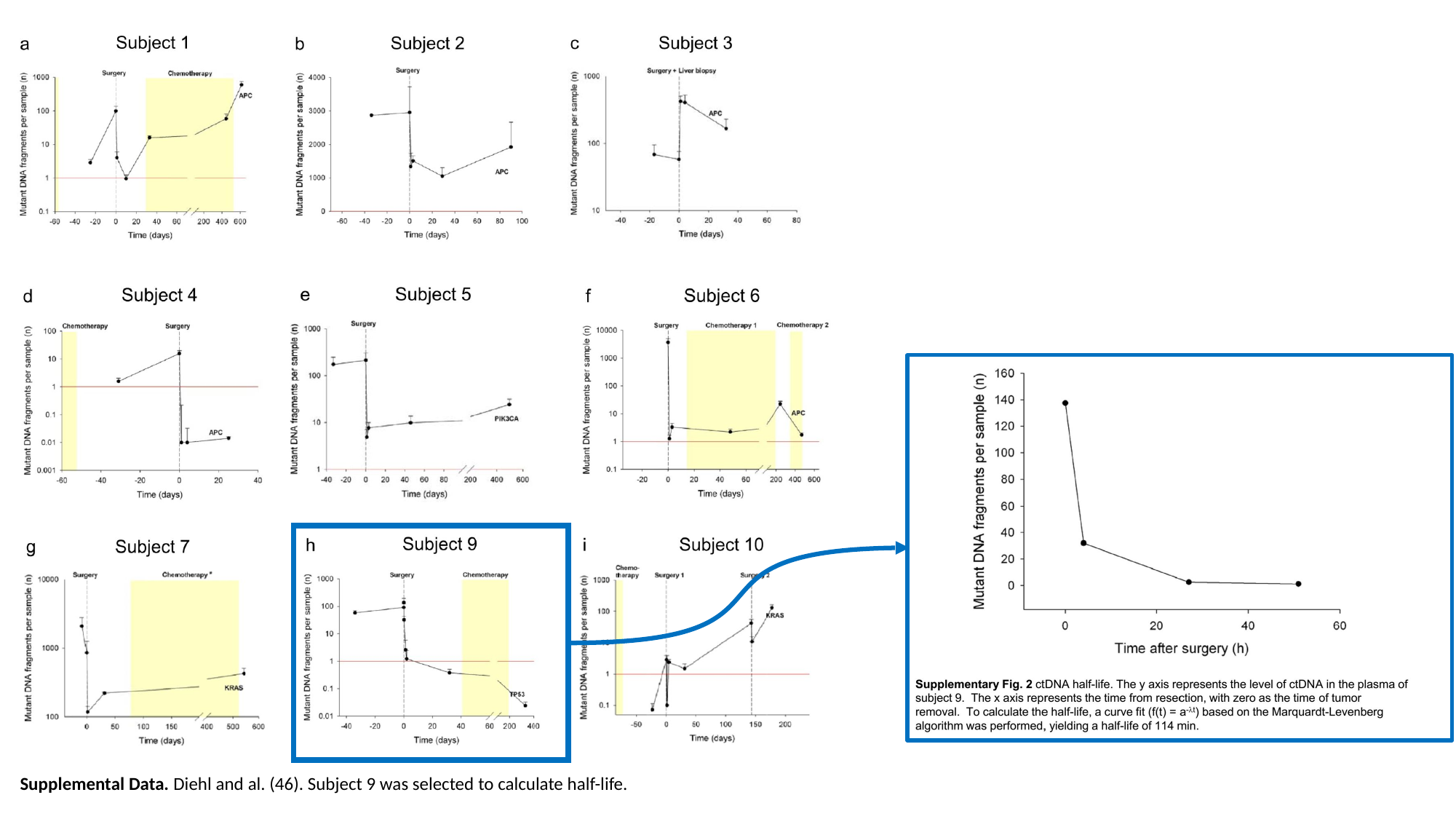

Supplemental Data. Diehl and al. (46). Subject 9 was selected to calculate half-life.

## Slide 3
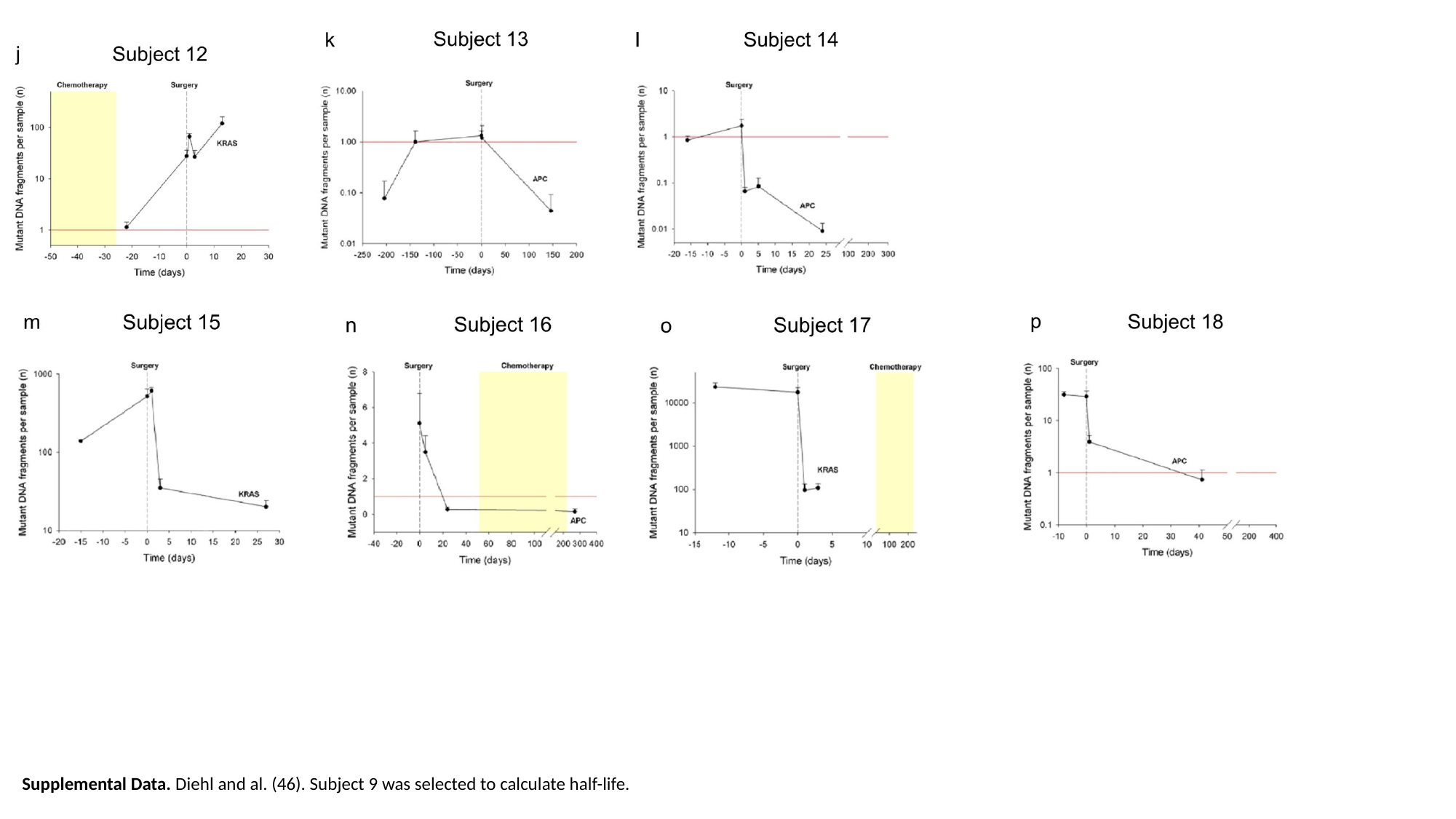

Supplemental Data. Diehl and al. (46). Subject 9 was selected to calculate half-life.

## Slide 4
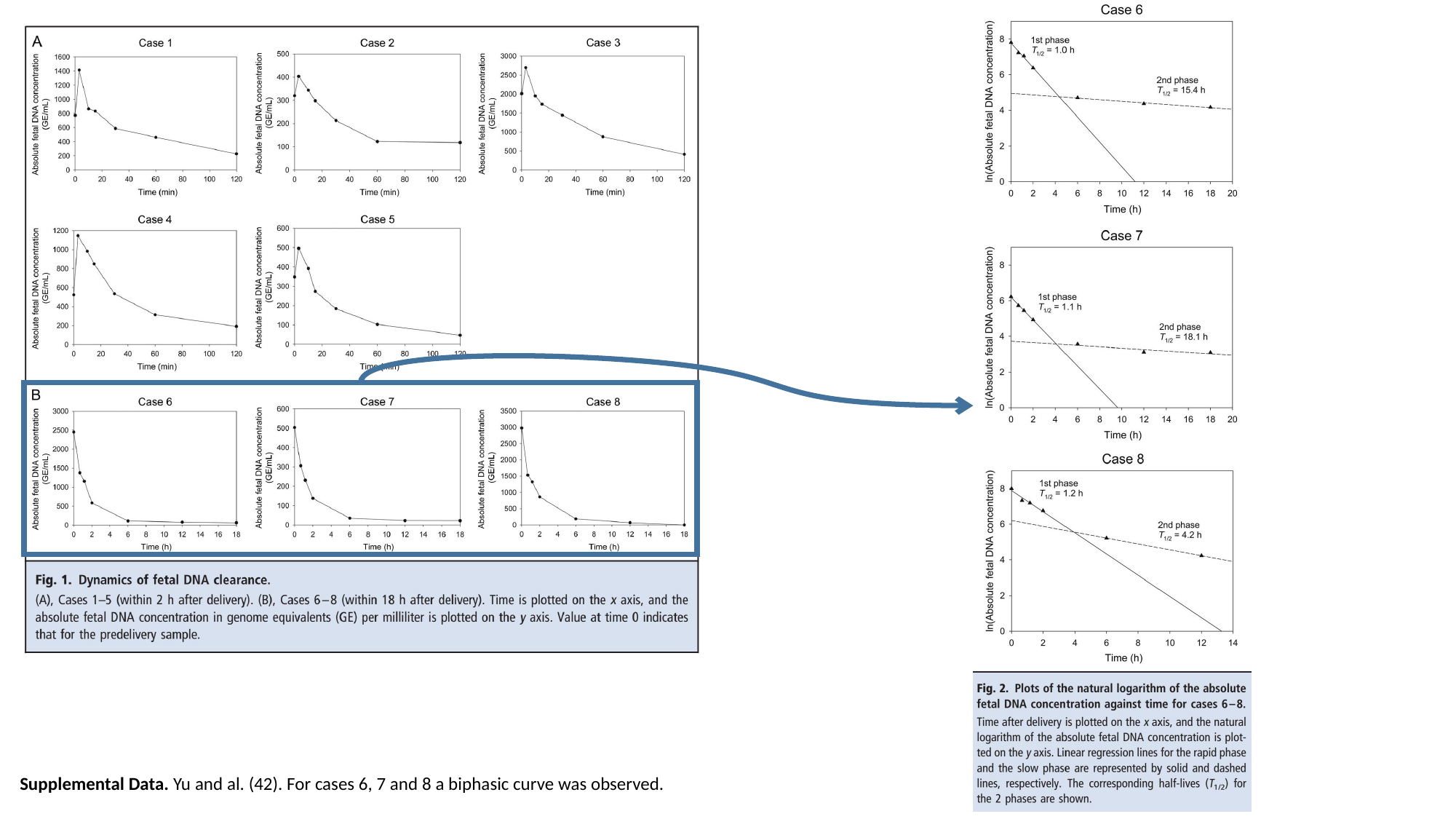

Supplemental Data. Yu and al. (42). For cases 6, 7 and 8 a biphasic curve was observed.

## Slide 5
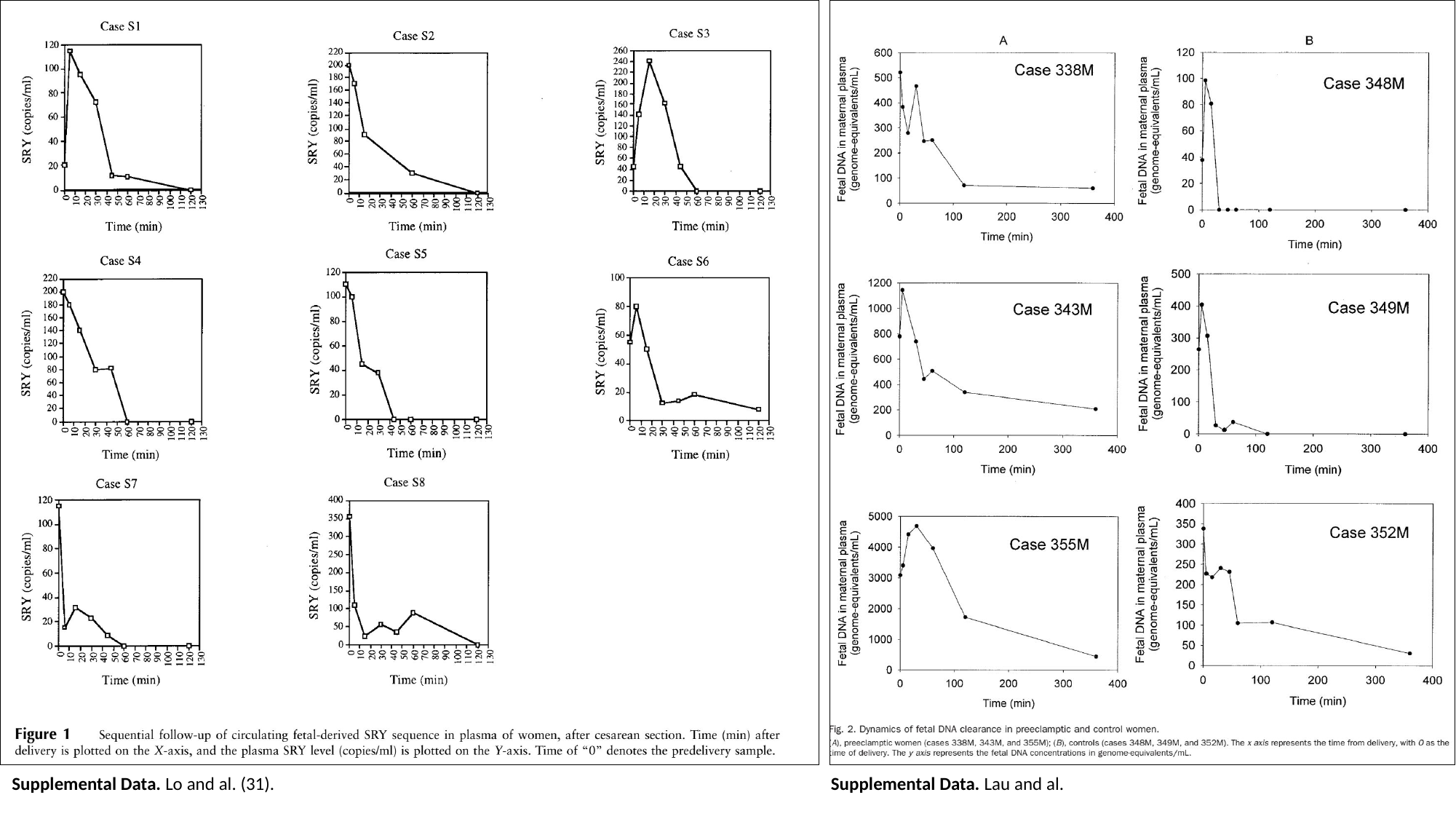

Supplemental Data. Lo and al. (31).
Supplemental Data. Lau and al.
