# Supplementary material for: Kinetics of circulating cell-free DNA for biomedical applications: critical appraisal of the literature
Source: Future Sci OA. 2018 Feb 23;4(4):FSO295. doi: 10.4155/fsoa-2017-0140 (PMC5905581; doi:10.4155/fsoa-2017-0140)
Supplement: Supplementary file 2 [file fsoa-04-295-s2.docx]

Supplemental Data:

# Selected studies : Method

We carried out a publication research through the PubMed library up to March 10, 2017. The following contextual query language was used: (“circulating” OR “cell-free” OR “plasma” AND “DNA” in the title) AND (“clearance” OR “kinetics” OR "half-life" in the text). Abstracts and text words were reviewed to identify reports assessing a kinetic parameter related to *stricto sensu* human cell-free DNA (e.g. endogenous human product). This led to “Humans” being ticked as a “Species” in the toolbar. Reference lists of identified studies and reviews were also hand-searched.

**Assess pharmacokinetic parameters (half-life of elimination)**

A pharmacokinetic (PK) analysis consist in studying time course of the drug concentration using mathematical models. It consists describe kinetic of distribution and elimination (and absorption if any) between different physiologic spaces named compartment by mathematical equations (Figure 1). The body is depicted as one or more compartments. Compartment is a hypothetical structure defined as a set of tissues, fluids or organs with similar kinetic characteristics (homogenous and instantaneous distribution in the compartment). Classical pharmacokinetic models result in mono- or multi-exponential equation ($C=f\left( t \right)$), which represent kinetic of the drug in the organism.

Based on $\int_{0}^{\infty} C_{(t)}.dt$, pharmacokinetic parameters could be estimated and each of these parameters characterize one step of kinetic. These PK parameters are : volume of distribution (1), half-life of distribution (t_λd_), clearance (2) and half-life of elimination (t_λz_) (3) etc. The half-life of elimination can be determined either directly from the terminal phase of $C=f\left( t \right)$ curve, or by employing the following relationship: $t_{\lambda z}= \frac{ln(2)}{\lambda z}$ (λz = slope of the log-linear terminal phase). t_λz_ is often called the biological half-life or terminal half-life. To obtain information about calculation of the other parameters, please consult (4). The usual way to explore terminal half-life (representing elimination process) is to apply a simple regression on the log-linear terminal curve. Based on assumption that after the last measured sample point (t_f_) the concentration declines as a mono-exponential. By means of log-linear regression of those observations, a slope is estimated fitting the last part of the curve, and a half-life could be estimated. Different way could be used to identify an appropriate range over which the linear regression could be done i) adjustment of the R-square: 3 last points for the linear regression and compute the R^2^ to this linear regression. Successively add earlier time points and make the linear regression on these data until R^2^ decreases etc. ii) use a mixt effect model on last part of the curve and apply a linear missed effect model iii) compute the derivative C’(t) at each point (5).

When the log representation of C=f(t) permits to describe two kinetic phases (two compartments model, 2 slopes) as showed in Figure 2, estimation of PK parameters is obtained by applying peeling method. It permits to not confuse half-life of distribution (t_λd_) and half-life of elimination (t_λz_). Application of the peeling method (or residuals method) will yield linear segment with slope and intercept from which the remaining value of λ_d_ and A_λd_ (6). Use compartmental pharmacokinetics modeling based on pharmacokinetic principles permits to describe all the sample points of kinetics by the simplest equation representative of the time course decrease. But models also permit to characterize more complex kinetics than a one phase decrease, and it is especially interesting when distribution phase(s) could be describe (Figure 2).

Software could also be used to analyze data. Different software exists to compute PK parameters. The half-live is compute by extrapolation and log-linear elimination process is assumed. That is, the terminal phase in the plot of log C_(t)_ *versus* time is approximately a straight line. Last part of the curve is assumed to be monoexponential. The slope and the intercept are estimated by least squares from the terminal phase. The time range included in the terminal phase could be specified by the user that is important because the user must be aware of the bias existing in protocol. Hence, he can upgrade the quality of analyze. If the user does not specify the terminal phase, an estimate from the best linear fit (based on adjusted R-square) will be used. Other parameters are compute in the same analyze, but also, the results of parameters values must be correctly interpreted.


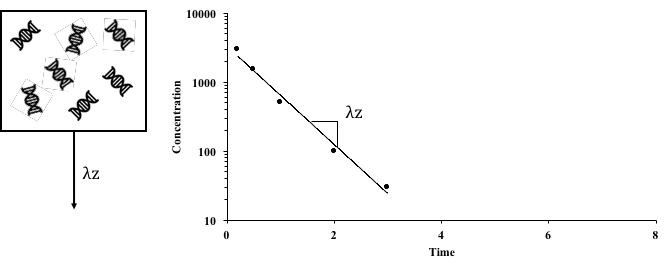


**Supplemental Data** Figure 1 : Mathematical scheme for a one compartment model

Distribution of ccf-DNA in the whole organism (e.g. one compartment = black box) with the same kinetic characteristics.

Concentration *versus* time decreases in the compartment as describe by the model :

$C_{(t)}=C_{\left( 0 \right)}.e^{-\lambda z.t}$ with a first order rate of elimination ($\lambda z$).

Pharmacokinetic parameter described by the model : biological HL *=* $t_{\lambda z}=\frac{\ln(2)}{\lambda z}$


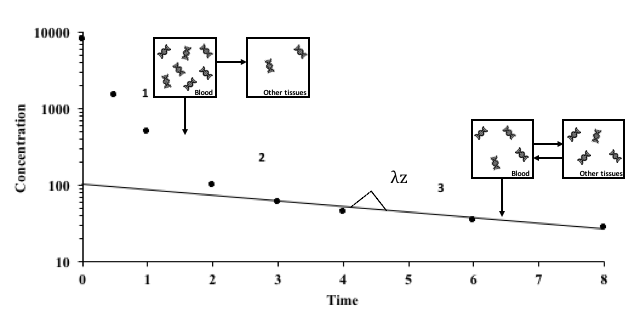


**Supplemental Data** Figure 2 : Mathematical scheme for a two compartments model

Distribution of ccf DNA in the organism with different kinetic characteristic between compartment (e.g. two compartments = black boxs).

Concentration *versus* time decreases in the blood compartment as describe by the model :

$C_{(t)}=A.e^{-\lambda d.t}+{B.e}^{-\lambda z.t}$ with a first order rate of distribution ($\lambda d)$ and elimination ($\lambda z$).

1: Decrease concentration from the blood due to distribution and elimination. 2: Inflexion point or pseudo-equilibrium state. 3: Decrease concentration from the blood due to elimination only (log-linear terminal phase).

Pharmacokinetic parameter described by the model : biological HL *=* $t_{\lambda z}=\frac{\ln(2)}{\lambda z}$

λ𝑧 is calculated directly from the terminal log-linear part of the curve, λd cannot be calculated directly from the curve, but by analytic the peeling method.

**References**

1. Toutain PL, Bousquet-Mélou A. Volumes of distribution. J Vet Pharmacol Ther 2004;27:441–53.

2. Toutain PL, Bousquet-Mélou A. Plasma clearance. J Vet Pharmacol Ther 2004;27:415–25.

3. Toutain PL, Bousquet-Mélou A. Plasma terminal half-life. J Vet Pharmacol Ther 2004;27:427–39.

4. Jambhekar SS, Breen P. Basic Pharmacokinetics. 2th Ed. Pharmaceutical Press; 2012.

5. Kallen A. Computational Pharmacokinetics. CRC Press; 2007.

6. Gibaldi M, Perrier D. Pharmacokinetics. 2th Ed. Taylor & Francis; 1982.
